# Adverse maternal and neonatal outcomes in Northern Sudan: the role of antenatal care and socioeconomic inequities in war

**DOI:** 10.1186/s12978-025-02129-4

**Published:** 2025-10-07

**Authors:** Suodad Elhassan, Marwa Osman, Ayat Altaher, Reem Mohamedalhassan Ahmed Mohamed, Saja Salah, Swsan Elsharif, Elshimaa Yousif

**Affiliations:** 1 Faculty of Medicine, University of Khartoum, Khartoum, Khartoum State Sudan; 2 Department of Community Medicine, Faculty of Medicine, University of Khartoum, Khartoum, Khartoum State Sudan; 3 Department of Public Health, Faculty of Medicine, Ankara Medipol University, Ankara, Turkey; 4 Faculty of Medicine, Merowe University of Technology, Merowe, Northern State Sudan; 5 Faculty of Medicine, Ahfad University for Women, Omdurman, Khartoum State Sudan

**Keywords:** Adverse maternal outcomes, Adverse neonatal outcomes, Birth outcomes, Maternal complications, Perinatal death, Antenatal care

## Abstract

**Background:**

The World Health Organization (WHO) has recommended increasing the minimum number of antenatal care (ANC) contacts from four to eight. High rates of maternal and neonatal complications and low ANC coverage are significant concerns in Sudan, especially after the war erupted. This study investigated the patterns of adverse maternal and neonatal outcomes and their correlation with ANC use and socioeconomic profile.

**Methods:**

This cross-sectional study was conducted on 267 postpartum women using convenience sampling in three public hospitals and one private clinic in Merowe locality, Northern Sudan. Structured interviews were conducted to collect data on socioeconomic and maternal characteristics, ANC use, and adverse maternal and neonatal outcomes. Descriptive, bivariate, and multivariate analyses were performed using Statistical Package for Social Science (SPSS).

**Results:**

The perinatal death rate was 3.4%. Preterm delivery and neonatal ICU admission rates were 10.1% and 7.1%, respectively. Adverse maternal outcomes occurred in 26.2% of the cases, with gestational hypertension (7.5%) and dystocia (4.1%) being the most prevalent. Displaced women showed higher rates of eclampsia and preeclampsia (*p* value = 0.002). Predictors of adverse maternal outcomes were gravidity of more than five (Adjusted Odds Ratio 2.55; 95% CI 1.04–6.23), rural residence (AOR = 2.52; 95% CI 1.25–5.12), and history of adverse events (AOR = 3.15; 95% CI 1.67–5.92). Fewer than four ANC contacts were associated with more perinatal deaths (*p* value = 0.032), while ≥ 8 visits did not improve the outcomes.

**Conclusions:**

The rate of adverse maternal outcomes was high and was influenced by obstetric and socioeconomic profiles. Attending at least four ANC visits was significantly associated with better birth outcomes. Addressing the social determinants of health and implementing screening programs for high-risk women are recommended.

**Supplementary Information:**

The online version contains supplementary material available at 10.1186/s12978-025-02129-4.

## Introduction

Maternal death and Severe Maternal Morbidity (SMM), are major public health concerns. Frequently considered indicative of a country’s health status, they often rise in developing nations. The world Maternal Mortality Ratio (MMR) was estimated to be 167 per 100,000 live births in 2023. Despite the 40% drop between 2000 and 2023, the World Health Organisation (WHO) described global maternal mortality in 2023 to be “unacceptably high”, mostly preventable, and mostly (92%) occurring in low- and middle-income countries (LMICs). Sub-Saharan Africa alone accounted for about 70% of these deaths, and countries with conflicts and fragility-including Sudan-accounted for 61%. This discrepancy reflects the gap between rich and poor, and inequalities in accessing healthcare [1, 2]. Causes of maternal deaths have a consistent theme across the developing world, according to the WHO systematic analysis, with direct obstetric complications causing 73% of maternal deaths and indirect causes accounting for the rest, mainly malaria, anaemia, and HIV [3]. In Sudan, MMR was estimated to be 256 in 2023, classified as moderate [4]. From 2000 to 2019, direct obstetric causes were responsible of 78.7% of maternal deaths, mainly due to haemorrhage (45.5%), Hypertensive disorders (16.1%), and sepsis (12.6%) [5].

Adverse maternal outcomes definition usually involves obstetric and non-obstetric conditions acquired during pregnancy, childbirth, or the immediate postpartum period, and most commonly include dystocia, haemorrhage, some modes of delivery (caesarean or vacuum extraction), hypertensive disorders of pregnancy, preterm or postdate pregnancy, anaemia, premature rupture of membrane (PROM), multiple pregnancy, and maternal death [6]. Certain factors increase the risk of adverse maternal outcomes, such as advanced maternal age (usually ≥ 35 years), comorbidities, disability, lack of antenatal care, and ethnicity [7, 8]. Allied to maternal outcomes, adverse neonatal outcomes include neonatal mortality and morbidity, such as low birth weight, preterm birth, admission to neonatal intensive care unit, stillbirth, or low APGAR score [9]. In Sudan, adverse pregnancy outcomes were linked to many factors, such as: grand multiparity, maternal undernutrition, anaemia, low Body Mass Index (BMI) and fewer ANC visits (less than two) [10–12]. Antenatal iron supplementation and ANC use were found to be protective against perinatal death, in contrast to home delivery and high parity (≥ 3) [13]. In Sudan Household Survey 2010, the rate of neonatal death was 3%, and was influenced by advanced maternal age, lower wealth, male sex, delivery by Caesarean Section (CS), and occurrence of obstetric complications [14].

The global community has adopted an approach focusing on the biomedical contribution of maternal mortality, paying less attention to other underlying determinants of maternal health, including social health and health systems. For instance, education, nutrition, and adequate access to effective healthcare play a crucial role in shaping maternal health, evidenced by clustering of deaths among the poorest populations [15, 16]. Several studies have linked lower wealth, rural residence, lower maternal education, and unemployment to higher incidences of adverse maternal and neonatal outcomes [17–19].

On the other hand, health system efficacy can act as a protective factor for women carrying risk factors at the individual level, such as age or socioeconomic factors, including antenatal, intrapartum, and postpartum care [15]. Therefore, the importance of early antenatal care emerges as an early predictor of prenatal complications and an identifier of high-risk pregnancies, especially when a delay in reaching medical help is expected, in addition to early detection and treatment of symptomless conditions, such as gestational hypertension, and asymptomatic bacteriuria (ASB), and provision of nutritional and social support [20].

After the introduction of WHO ANC model in 2002 (focused ANC model), ANC utilization increased in LMICs; however, early antenatal care coverage was found to be only 24% in Low- income countries (LICs), compared to 81.9% in countries with high income [20, 21]. The WHO has recommended at least eight ANC visits, instead of four, especially in LICs, with the first visit being in the first trimester [20]. Several measures are applied to attain the aforementioned goals, including: ultrasound, urine examination, vaccination, screening and management of preeclampsia, gestational diabetes, anaemia, addressing mental and social health, and nutritional counselling [20, 22].

In the context of armed conflict, displacement and service interruptions may jeopardize maternal health. The highest newborn mortality rates occurred in fragile settings and nations that have suffered a recent humanitarian catastrophe [23]. However, trends in adverse maternal and neonatal outcomes remain under investigated. The huge internal displacement flow in Sudan has redistributed a considerable percentage of the population disproportionately to the readiness of the health system, compromising access to essential services. Yet, little is known about the new situation forced by the war. This study aimed to identify patterns of adverse maternal and neonatal outcomes in relation to maternal and socioeconomic characteristics, displacement, and ANC use, and to determine predictors of poor maternal outcomes in Merowe, Northern Sudan, therefore, providing basis for tailored interventions to support maternal health and identify women at risk.

## Materials and methods

### Study design and setting

This was a descriptive, cross-sectional, facility-based study conducted among postpartum women in Merowe locality in northern Sudan. After the eruption of the war on April 2023, the Northern State has received more than 400,000 internally displaced people by March 2024 [24]. Merowe area is predominantly rural, with the area’s economy revolving around agriculture, pastoralism, and river-based livelihoods. The completion of the Merowe dam in 2009 and the subsequent displacement were thought to cause social and economic disruption to some local communities, but in overall, it improved living conditions in terms of food production, electricity, and water supply [25]. While maternal health data specific to Merowe are unavailable, the Northern state has shown a significant decline in the MMR from 2009 to 2019. However, its MMR (131.27) remained classified as moderate according to the WHO, and higher than most other states [5].

The study area included two major towns in the locality: Merowe and Karima. Common delivery sites include four secondary and tertiary hospitals, private clinics, primary healthcare facilities, and home deliveries.

### Study population

The study included Sudanese postpartum women attending the selected facilities between 17th July 2024 and 2nd October 2024, whether they delivered in this facility or elsewhere. We excluded women with severe psychological distress or psychiatric disorders, communication barriers, and women whose pregnancy outcome was fetal death before the 28th week of gestation.

### Sampling technique and sample size

Merowe locality contains numerous facilities for delivery, including distributed healthcare centres, few rural hospitals, and central referral hospitals. In this study, we focused on a sample frame containing the four highest-volume facilities:

1. Merowe General Hospital (a public secondary hospital).
2. Merowe Military Hospital (a public secondary hospital).
3. Karima Teaching Hospital (a public tertiary hospital).
4. Al-Rayan Private Clinic (one of the largest private providers).

These facilities collectively handle most facility births in Merowe and receive the highest rates of obstetrics patients. They also serve as referral facilities for pregnant women from remote and underserved areas.

We used nonprobability convenience sampling to select patients. This sampling approach was necessitated by logistical constraints (staff availability, security during floods, and conflict-related travel restrictions). Postpartum women were identified from hospital logs and approached. All the available postpartum women who met the aforementioned inclusion criteria were included consecutively at the time of data collection. The sample size was calculated using Cochran’s formula, with a level of precision (e) = 0.05 (5%), confidence interval (z) = 1.96 (95%), and proportion of adequate antenatal care coverage (≥ four contacts) (p) = 0.11 [26]. The calculated sample size (n) was 151 women, representing the minimum required to detect the outcome. A total of 267 women were included in this study to be able to carry out multivariate regression which needs 10–15 events per predictor variable.

### Data collection methods and tools

We used a pretested, author-designed, closed-ended questionnaire to collect data on birth, maternal and neonatal outcomes, socioeconomic profile, obstetric history, and use of antenatal care (ANC) services. The questionnaire was reviewed and validated by an epidemiologist, two physicians, and an obstetrics and gynaecology registrar. A pilot study was conducted by distributing 30 questionnaires in the same study settings to test the feasibility and validity of the questionnaire, and any expected human-related and data quality challenges at the facilities; consequently, a more appropriate and explanatory language was adopted, and the questions sequence was modified to enhance logical flow. The final version of the questionnaire was approved by the experts. Women were interviewed by medical practitioners who were trained on the use of the questionnaire, and who were fluent in the local language. Consistency and standardisation were further ensured by educating data collectors on the clinical and operational definitions of terms used in the survey and by using an electronic data entry system (Kobo Toolbox) with built-in logic and validity checks. Outcomes and clinical data were cross-checked with the patients’ medical records.

A participant was considered to have an adverse maternal outcome if they experienced any complication related to this most recent pregnancy, delivery, or immediate postpartum stage, including dystocia, preterm and premature rupture of membrane (PROM), gestational diabetes and hypertension, antepartum and postpartum haemorrhage, eclampsia and pre-eclampsia, placental abruption, maternal sepsis, chorioamnionitis, uterine rupture, ICU admission, and death. Neonatal adverse outcomes include: peri-natal mortality, NICU admission, pre-term delivery, and any neonatal morbidity related to pregnancy or delivery (birth trauma, respiratory distress, aspiration and asphyxia, low birth weight, being small or large to gestational age, sepsis, etc.)

We applied the International Classification of Diseases (ICD) operational case definitions and criteria to identify adverse outcomes [27]. In this study, perinatal death term did not include early stillbirth (22nd–27th weeks) as the age of fetal viability is considered 28 weeks of gestation in Sudan.

### Data management and analysis

The Data were coded and assigned numbers according to a pre-prepared codebook. Data cleaning, refinement, and analysis were performed using the Statistical Package for Social Science (SPSS) version 21 software.

Descriptive statistics were presented as frequencies (n), percentages (%), means, and standard deviations (SD). The relationship between adverse outcomes and socioeconomic profile, maternal characteristics, and ANC use was assessed using Chi-square test and Fisher’s exact test. Multivariate logistic regression was used to identify the predictors of adverse maternal outcomes. Statistical significance was set at *P* < 0.05.

### Ethical considerations

This study was conducted in accordance with the ethical principles outlined in the Declaration of Helsinki. Ethical approval was granted by the Department of Community Medicine, Faculty of Medicine, University of Khartoum [ID: COMMED 2024-95-23]. Permission was granted by the administration of each facility.

Informed consent was obtained from each woman after explaining the study objectives and the type of information to be collected. Additional consent was granted from a caregiver for patients below the age of 18 years.

## Results

### Characteristics of the study participants and war effects

The study included 267 postpartum women who gave birth in Merowe locality in the Northern State. The age range was 16–44 years, with a mean age of 28 years. Most women (66.7%) settled in rural settings. Nearly one-third (31.5%) lived in extended family homes rather than independently. Less than half of the women were covered by health insurance, mainly under social insurance scheme (Table 1).

**Table 1:** Socio-economic characteristics of postpartum women at Merowe locality, Northern State, Sudan 2024

Variable	Sub-category	Frequency	Percentage
Age	30 or less	180	67.4
	More than 30	87	32.6
	Mean (SD)	28.4 (5.7)	
Marital status	Married	267	100
	Divorced or widowed	0	0
Residence	Urban	88	33.0
	Rural	178	66.7
	Missing	1	0.4
Education	Illiterate	17	6.4
	Primary school	65	24.3
	Secondary school	68	25.5
	University or above	117	43.8
Occupation	Housewife	227	85.0
	Formal job	36	13.9
	Self-employed or informal worker	3	1.1
Husband Occupation	Formal Job	57	21.3
	Self-employed or informal worker	198	74.2
	Non-working	11	4.1
	Missing	1	0.4
Health Insurance	Social Health Insurance (SHI)	95	35.6
	Private	13	4.9
	No insurance	159	59.6
Housing	Owned house	167	62.5
	Rented house	15	5.6
	With extended family or relatives	84	31.5
	Displacement camp	1	0.4
Household size	≤ 6	165	61.8
	≥ 7	102	38.2
	Median (IQR)	6 (4)	
Displacement	Yes	62	23.2
	No	205	76.8
Duration of displacement	≤ 12 months	33	53.2
	> 12 months	29	46.8
	Mean (SD)	11.1 (4.9)	

n = 267

Twenty-three percent of women were displaced, mostly from Khartoum State (85.5%). About 6.5% of women reported that they lost a fetus or neonate due to the ongoing war, and 8.8% of displaced families lost their breadwinners. Only 12.9% of displaced women received humanitarian aid.

### Obstetric and clinical profile

Caesarean section was the major delivery mode (78.3%), and multifetal pregnancy rate was 4.1%. The gestational age ranged from 30 to 42 weeks, with an average of 38 weeks. Gravidity was as high as 11 in some women, with a median of three (interquartile range = 2–4).

The majority (93.3%) had no chronic comorbidities, but 1.9% had chronic hypertension, 3.7% had chronic diabetes, and 0.4% had chronic heart disease.

### Birth outcomes and adverse neonatal outcomes

**Fig. 1:**
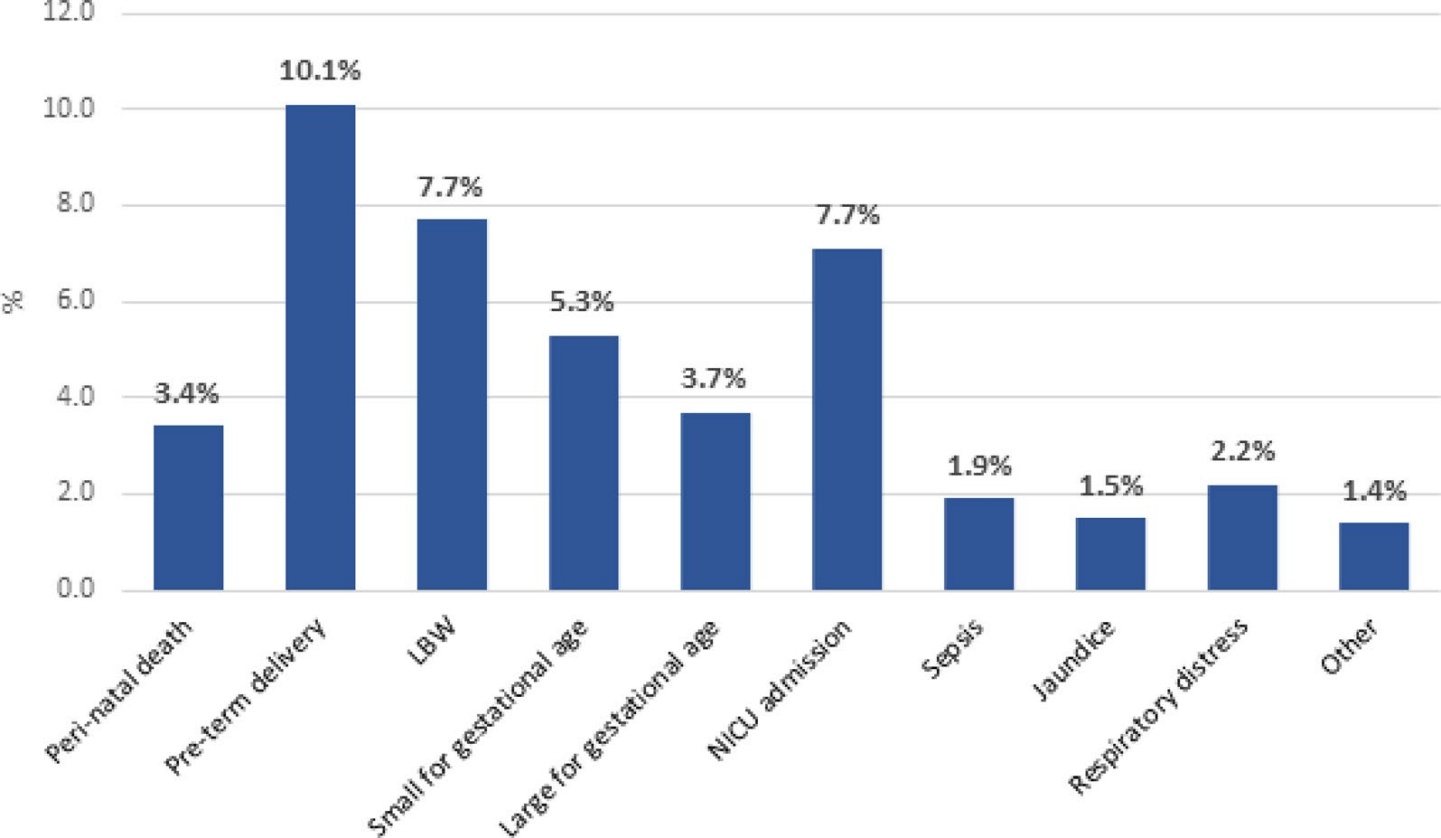
Adverse neonatal outcomes at Merowe locality, Northern state, Sudan 2024. n = 267

The perinatal mortality rate was 3.4%, while 10.1% of the neonates were delivered preterm (before the 37th week of gestation). Approximately 7.1% were admitted to the NICU. Respiratory distress, sepsis, and jaundice were present in 2.2%, 1.9%, and 1.5% of neonates, respectively. Other reported neonatal morbidities included convulsions, fever of unknown origin, intestinal obstruction, and bleeding (1.4%) (Fig. 1).

Weight data were available for approximately half of the neonates (N = 130), ranging from 1.4 to 5.5 kg, and the average birth weight was 2.9 kg (SD = 0.6). LBW was present in 7.7% of the neonates weighed (Fig. 1).

### Adverse maternal outcomes

**Fig. 2:**
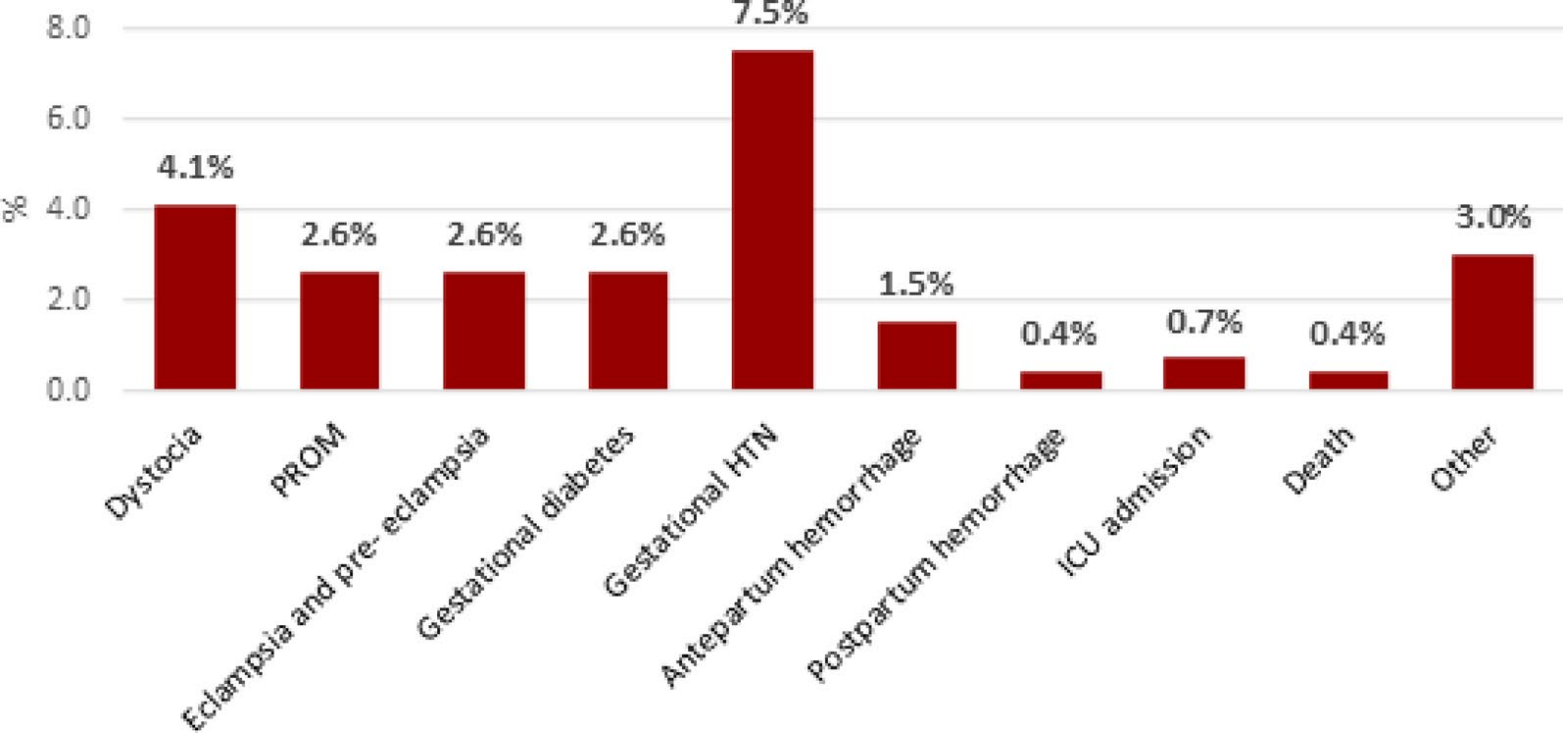
Adverse maternal outcomes at Merowe Locality, Northern state, Sudan 2024. n = 267

The overall rate of adverse maternal outcomes was 26.2% (excluding multigravidity). The most frequently recorded outcome was gestational hypertension (7.5%). A total of 4.1% of postpartum women experienced dystocia. The rates of other adverse maternal outcomes are presented in Fig. 2. Other maternal outcomes included oligohydramnios and polyhydramnios (1.9%), placental abruption (0.4%), uterine rupture (0.4%), and DKA (0.4%) (Fig. 2).

We found that a large proportion of women (43.1%) suffered from urinary tract infections (UTI) during pregnancy, nearly one-fifth reported incidence of malaria, and 9.7% had anaemia.

### Determinants of adverse maternal and neonatal outcomes

Older women showed a significantly higher incidence of adverse maternal outcomes; the overall incidence was 34.5% among women older than 30 years compared to only 22.2% among younger women (*p* value = 0.047). Incidence was also associated with higher gravidity (> five) (*p* value = 0.009), rural residence (*p* value = 0.013), and larger households (*p* value = 0.026).

Eclampsia and preeclampsia were influenced by displacement, poor housing, larger households, and women’s occupation (*p* value < 0.05) (Supplementary Table 1).

**Fig. 3:**
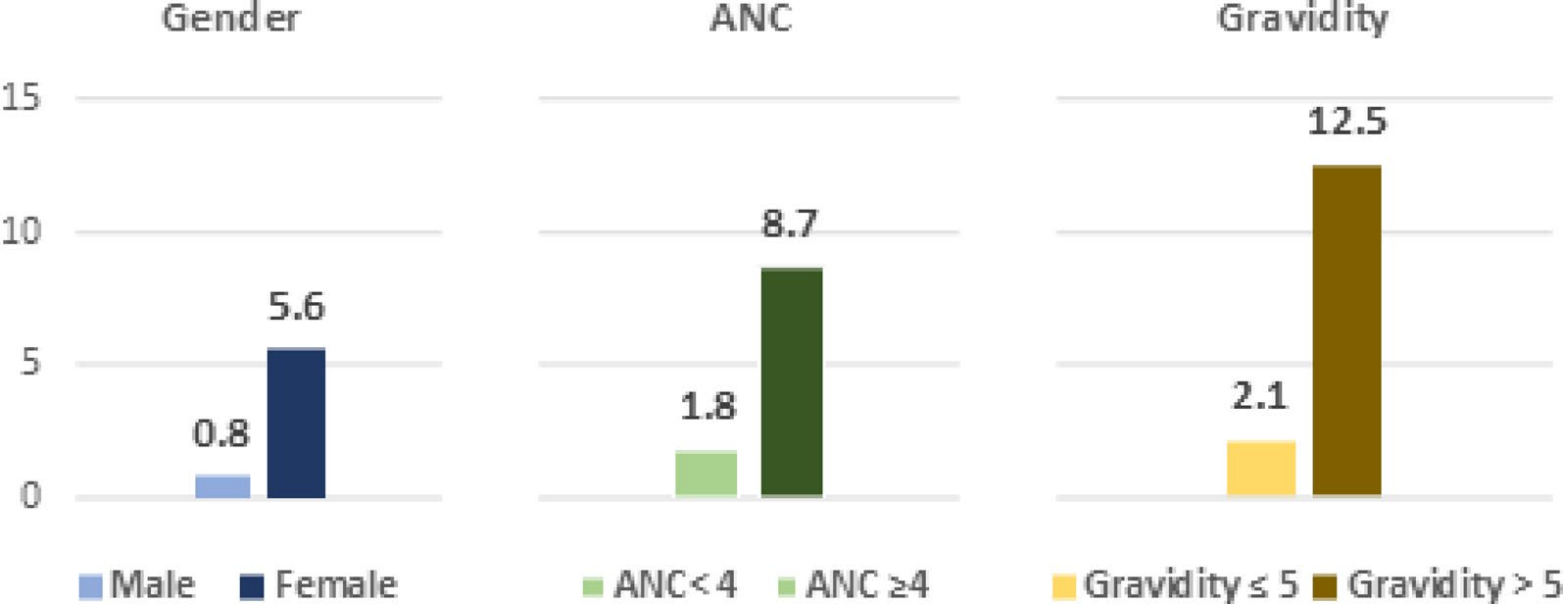
Perinatal death rate according to gender, antenatal care, and gravidity at Merowe Locality, Northern state, Sudan 2024. n = 267

The perinatal death rate was significantly higher with gravidity > 5 (12.5%) than with lower gravidity (2.1%) (*p* value = 0.014). Moreover, it was also higher in female neonates (5.6%) than in male neonates (0.8%) after excluding multiple pregnancies (*p* value = 0.032) (Fig. 3). Higher rates of adverse neonatal outcomes were observed in women living in larger households (Supplementary Table 2).

### Impact of ANC utilization on maternal and neonatal outcomes

We found that 99.3% of women attended ANC at least once during their pregnancy, 82.4% attended at least four visits, and 25.8% attended eight visits or more.

Women with fewer than four ANC visits had significantly higher rates of perinatal death (8.7% vs. 1.8% in those with ≥ 4 visits; *p* value = 0.032) (Fig. 3). However, achieving the recommended minimum of four ANC visits or the optimal eight visits did not significantly reduce the overall rates of adverse maternal or neonatal outcomes (*p* value > 0.05) (Supplementary Table 3).

### Predictors of adverse maternal outcomes using binary logistic regression

Logistic regression was performed to assess the effects of six variables on the incidence of adverse maternal outcomes. The model was statistically significant, explaining 15.6% of the variability in incidence (Nagelkerke R^2^ = 0.156) and correctly classifying 74.6% of the cases.

Women residing in rural areas were 2.5 times more likely to experience adverse outcomes than those in urban areas (AOR = 2.527, 95% CI = 1.248–5.117, *P* value = 0.010). Obstetric profiles played a significant role in predicting adverse outcomes; women with a history of previous adverse maternal outcomes had approximately three times higher odds of experiencing another event (AOR = 3.145, 95% CI = 1.670–5.922, *P* value < 0.001). Similarly, high gravidity (> 5) was associated with 2.5 times higher odds of experiencing an adverse event (AOR = 2.546, 95% CI = 1.041–6.228, *P* value = 0.041) (Table 2).

**Table 2:** Logistic regression for predictors of adverse maternal outcome incidence outcomes in Merowe Locality, Northern state, Sudan 2024

Variable	B	S.E	*P* value	Odds ratio	OR (95% CI)	
Age above 30 (ref; ≤ 30 yrs)	0.303	0.354	0.391	1.354	0.677	2.710
Gravidity >5 (ref; ≤ 5)	0.935	0.456	0.041*	2.546	1.041	6.228
Rural residence (ref; urban)	0.927	0.360	0.010*	2.527	1.248	5.117
Multi-fetal pregnancy	1.200	0.682	0.079	3.319	0.872	12.630
History of adverse maternal outcomes	1.146	0.323	< 0.001*	3.145	1.670	5.922
At least 4 ANC visit (ref; less than 4 visits)	− 0.424	0.392	0.280	0.654	0.303	1.412

n = 267

B: Effect Estimate; S.E: standard error; CI: confidence interval

In contrast, low ANC attendance (< 4 visits), age, and multifetal pregnancy did not predict adverse maternal outcomes (*p* value > 0.05).

## Discussion

Ongoing armed conflicts in Sudan have fragmented the healthcare system and hindered access to essential services [28]. This disruption, in addition to the vulnerability precipitated by displacement, is expected to jeopardise maternal and neonatal health [29], especially with the high maternal and neonatal mortality pre-war [30]. This study aimed to identify patterns of adverse maternal and neonatal outcomes, and explore the effect of ANC use and other socioeconomic and maternal factors in a semi-rural community that received a large displacement flow.

The results showed that women from rural residential settings predominated (66.7%), which was expected. More than half of the participants (59.6%) did not have health insurance. This may have its effect on encouraging hospital delivery and ANC use. Nearly 30% do not own or rent their own houses; rather, they reside with relatives or extended family houses or displacement camps. This is another indicator of the instability imposed by displacement. The displaced population constituted about one-fourth of the sample. In Sudan, poorer maternal outcomes were linked to lower wealth, rural residence, and illiteracy [13, 14, 31]. Studies have shown that low socioeconomic status (SES) correlates with higher rates of stillbirth, perinatal mortality, and poor maternal outcomes in LMICs [32, 33] and suggested that obtaining higher levels of education and better living standards may minimise maternal and neonatal morbidity [34].

The war’s eruption resulted in the largest displacement crisis globally [35]. Displaced women often face several challenges and are more vulnerable to malnutrition and infectious diseases. In this study, eclampsia and preeclampsia were linked to displacement; the rate of this outcome was 9.7% in displaced women compared to only 1.0% in non-displaced women. While there is no previously established link between eclampsia and displacement in the literature, lower SES has been previously identified as a risk factor for eclampsia and preeclampsia [18, 36, 37]. We believe that displacement may exacerbate some risk factors such as socioeconomic challenges, reduced and late access to care, and anaemia [38, 39].

The most prevalent maternal outcomes in this study were gestational HTN (7.5%) and dystocia (4.1%), followed by premature rupture of membrane, gestational diabetes, and eclampsia/ preeclampsia (2.6%). In our study, we found higher rates of preterm labour (10.1%), NICU admission (7.1%), and dystocia (4.1%) compared to eastern Sudan, where only 2.6% of neonates were delivered preterm, 6% were admitted to NICU, and only 1.9% of women had dystocia [40].

Malaria (18.7%) and UTI (43.1%) were highly prevalent. A study in central Sudan found that asymptomatic bacteriuria was present in 13% of pregnant women [41]. Accordingly, effective ANC is of great value in the detection of hidden diseases and/or early treatment of conditions that emerge during pregnancy. Several studies in Sudan have linked malaria to poor obstetric outcomes; pregnancy malaria was associated with anaemia during pregnancy and stillbirth and was responsible for 11.5% of maternal mortality [39, 40]. Preeclampsia was also associated with placental malaria infection [42].

This study found that perinatal death rate was only 1.8% in women with four or more ANC visits, compared to 8.7% in women with fewer visits (Fig. 3). Adequate antenatal care allows for the early detection and management of pregnancy conditions, fetal monitoring, and preventive interventions. Several studies have recognised inadequate ANC as a risk factor for perinatal death [43–47]. Some studies found higher perinatal mortality among women who did not attend any ANC compared to those who attended at least one visit [44, 45, 47]. Other studies have also identified a cutoff point of four visits as a protective factor against perinatal death [43, 46, 48]. In our study, the coverage of the first and fourth ANC visits was quite high (99.3% and 82.4%, respectively), whereas it was 90% and 11% in eastern Sudan, respectively [26]. Interestingly, female neonates had a higher rate of perinatal death than that of male neonates. Studies in India have found that female neonates and infants have a higher mortality rate [49, 50]. Other studies in Pakistan and Japan found that male neonates had higher rates of adverse outcomes and mortality [51, 52].

Women with gravidity > 5 had 2.5 times higher odds of experiencing adverse maternal outcomes. This finding aligns with the existing literature, suggesting that grand multigravidity and multiparity predispose women to maternal complications, including malpresentation, haemorrhage, preterm delivery, gestational HTN, and neonatal ICU admission [12, 53, 54]. Interestingly, suboptimal ANC utilisation did not emerge as a significant predictor of adverse outcomes, although previous studies have linked poor antenatal care to adverse labour outcomes [31, 55, 56]. A secondary analysis of the WHO Antenatal Care Trial comparing the standard ANC model with the package of reduced visits has linked the reduced visits model to a higher relative risk of fetal death at less than 36 weeks in both low-risk and high-risk women; however, it also suggested that differences in settings, timing, and quality of visits could play a role. Therefore, monitoring maternal and fetal outcomes when implementing ANC protocols is essential [57]. For instance, a study in Ethiopia found that average-to-poor-quality ANC predicted anaemia, severe preeclampsia, and LBW [58]. Both rural residence and previous maternal complications predicted adverse outcomes in the current pregnancy, similar to other studies [17, 59, 60]. Rural residence may exert an effect through the socioeconomic determinants of health and lower access to care. This reflects the need for proper identification of high-risk pregnancies based on obstetric history, in addition to screening programs, addressing the social determinants of health, and strengthening the rural health system by improving transport and referral systems and training and engaging village midwives in risk detection and emergency response. Remarkably, in Sudan, and based on the three delays model, maternal deaths due to the delay at home and the delay in reaching the hospital were significantly more prominent [5].

This is a novel study in this geographical location, as there is a lack of data on the status of ANC use and maternal and neonatal health. We focused on including both socioeconomic and clinical factors to provide a more comprehensive understanding of maternal risk factors. One of the limitations of this study is the low representation of home deliveries, as data were collected from labour rooms and inpatient wards. Due to the lack of data in this region, further community-based longitudinal studies are recommended. While the non-probability sampling approach may limit generalisability, consecutive enrolment minimised selection bias, and the expanded sample size improved statistical precision. Future studies should explore more variables, such as nutritional status and ANC quality.

## Conclusion

The study revealed a rate of 26.2% adverse maternal outcomes, with 82.4% of the women attending at least four ANC visits. Women receiving fewer than four ANC contacts had significantly worse birth outcomes, specifically 8.7% perinatal mortality, compared to only 1.8% among those with ≥ 4 contacts (*p* < 0.05). Rural residence, high gravidity, and a history of maternal complications predicted adverse maternal outcomes. Eclampsia/preeclampsia were significantly associated with the socioeconomic status of the respondent, namely displacement, housing, and large household size. We recommend screening programs to identify high-risk pregnancies, addressing social determinants of maternal health, and strengthening rural health system by improving referral and midwife training in risk identification and emergency recognition and response.

## Supplementary Information

Additional file1 (DOCX 29 kb)

## Data Availability

Data supporting these findings are available from the corresponding author upon reasonable request.

## Abbreviations

ANC: Antenatal care
BMI: Body mass index
CS: Caesarean section
HTN: Hypertension
ICD: International Classification of Diseases
LBW: Low birth weight
LMICs: Low- and middle-income countries
MMR: Maternal mortality ratio
NICU: Neonatal intensive care unit
PROM: Premature rupture of membrane
SES: Socioeconomic status
SHI: Social health insurance
SMM: Severe maternal morbidity
UTI: Urinary tract infection
WHO: World Health Organisation
