# Supplementary material for: Adverse maternal and neonatal outcomes in Northern Sudan: the role of antenatal care and socioeconomic inequities in war
Source: Reprod Health. 2025 Oct 7;22:186. doi: 10.1186/s12978-025-02129-4 (PMC12502332; doi:10.1186/s12978-025-02129-4)
Supplement: Supplementary file 1 — Additional file1 (DOCX 29 kb) [file 12978_2025_2129_MOESM1_ESM.docx]

# Supplementary Materials

Supplementary Table 1. Association between Maternal and Socio-economic characteristics and Adverse maternal Outcomes Incidences at Merowe Locality, Northern state, Sudan 2024.  n=267

| **Variable** | **Sub-category** | **Incidences of advanced maternal outcome (%)** | | **P-value** | **Eclampsia and preeclampsia (%)** | | **P-value** |
| --- | --- | --- | --- | --- | --- | --- | --- |
|  |  | Yes | No |  | Yes | No |  |
| **Maternal age** | Up to 30 | 40 (22.2) | 140 (77.8) | 0.047* | 6 (3.3) | 174 (96.7) | 0.726 |
|  | Above 30 | 30 (34.5) | 57 (65.5) |  | 2 (2.3) | 85 (97.7) |  |
| **Gravidity** | Up to 5 | 55 (23.5) | 179 (76.5) | 0.009* | 6 (2.6) | 228 (97.4) | 0.248 |
|  | More than 5 | 15 (46.9) | 17 (53.1) |  | 2 (6.3) | 30 (93.8) |  |
| **Residence** | Urban | 14(15.9) | 74 (84.1) | 0.013 * | 1 (1.1) | 87 (98.9) | 0.880 |
|  | Rural | 55 (30.9) | 123 (69.1) |  | 7 (3.9) | 171 (96.1) |  |
| **Education level** | Illiterates | 6 (35.3) | 11 (64.7) | 0.558 | 0 (0) | 17 (100) | 0.892 |
|  | Primary school | 20 (30.8) | 45 (69.2) |  | 3 (4.6) | 62 (95.4) |  |
|  | Secondary school | 17 (25.0) | 51 (75.0) |  | 2 (2.9) | 66 (97.1) |  |
|  | University or above | 27 (23.1) | 90 (76.9) |  | 3 (2.6) | 114 (97.4) |  |
| **Occupation** | Housewife | 57 (74.9) | 170 (25.1) | 0.519 | 4 (1.8) | 223 (98.2) | 0.009* |
|  | Formal Job | 12 (32.4) | 25 (67.6) |  | 3 (8.1) | 34 (91.9) |  |
|  | Informal worker/ self employed | 1 (33.3) | 2 (66.7) |  | 1 (33.3) | 2 (66.7) |  |
| **Husband Occupation** | Informal worker/ self employed | 50 (25.3) | 148 (74.7) | 0.652 | 4 (2.0) | 194 (98.0) | 0.096 |
|  | Formal job | 17 (29.8) | 40 (70.2) |  | 3 (5.3) | 54 (94.7) |  |
|  | Non-working | 2 (18.2) | 9 (81.8) |  | 1 (9.1) | 10 (90.0) |  |
| **Displacement** | Yes | 17 (27.4) | 45 (72.6) | 0.936 | 6 (9.7) | 56 (90.3) | 0.002* |
|  | No | 53 (26.9) | 152 (74.1) |  | 2 (1.0) | 203 (99.0) |  |
| **Displacement duration** | Up to a year | 12 (36.4) | 21 (63.6) | 0.162 | 5 (15.2) | 28 (84.8) | 0.201 |
|  | More than a year | 5 (17.2) | 24 (82.8) |  | 1 (3.4) | 28 (96.6) |  |
| **Housing** | Owned house | 48 (28.7) | 119 (71.3) | 0.079 | 2 (1.2) | 165 (98.8) | 0.030* |
|  | Rented house | 6 (40.0) | 9 (60.) |  | 2 (13.3) | 13 (86.7) |  |
|  | Family or Relatives house | 15 (17.9) | 69 (82.1) |  | 3 (3.6) | 81 (96.4) |  |
| **Household size** | Up to 6 | 35 (21.2) | 130 (78.8) | 0.026* | 2 (1.2) | 163 (98.8) | 0.037* |
|  | More than 6 | 35 (34.3) | 67 (65.7) |  | 6 (5.9) | 96 (94.1) |  |
| **Health Insurance** | None | 43 (27.0) | 116 (73.0) | 0.822 | 6 (3.6) | 153 (96.2) | 0.809 |
|  | SHI | 23 (24.2) | 72 (75.8) |  | 2 (2.1) | 93 (97.9) |  |
|  | Private | 4 (30.8) | 9 (69.2) |  | 1 (0.0) | 13 (100) |  |

* Association is significant at the level 0.05 (2-tailed)

Supplementary Table 2. Determinants of Perinatal death Adverse Neonatal Outcomes Incidence at Merowe Locality, Northern state, Sudan 2024.  n=267

| **Variable** | **Sub-category** | **Incidences of advanced neonatal outcome (%)** | | **P-value** | **Peri-natal death (%)** | | **P-value** |
| --- | --- | --- | --- | --- | --- | --- | --- |
|  |  | Yes | No |  | Yes | No |  |
| **Neonate Gender*** | Male | 18 (13.7) | 113 (86.3) | 0.382 | 1 (0.8) | 130 (99.2) | 0.032 |
|  | Female | 23 (18.5) | 101 (81.5) |  | 7 (5.6) | 117 (94.4) |  |
| **Maternal age** | Up to 30 | 29 (16.1) | 151 (83.9) | 0.454 | 4 (2.2) | 176 (97.8) | 0.130 |
|  | Above 30 | 18 (20.7) | 69 (79.3) |  | 5 (5.7) | 82 (94.3) |  |
| **Gravidity** | Up to 5 | 38 (16.2) | 196 (83.8) | 0.160 | 5 (2.1) | 229 (97.9) | 0.014 |
|  | More than 5 | 9 (28.1) | 23 (71.9) |  | 4 (12.5) | 28 (87.5) |  |
| **Residence** | Urban | 12 (13.6) | 76 (86.4) | 0.349 | 2 (2.3) | 86 (97.7) | 0.380 |
|  | Rural | 34 (19.1) | 144 (80.9) |  | 7 (3.9) | 171 (96.1) |  |
| **Education level** | Illiterates | 5 (29.4) | 12 (70.6) | 0.540 | 1 (5.9) | 16 (94.1) | 0.700 |
|  | Primary school | 11 (16.9) | 54 (83.1) |  | 2 (3.1) | 63 (96.9) |  |
|  | Secondary school | 13 (19.1) | 55 (80.9) |  | 3 (4.4) | 65 (95.6) |  |
|  | University or above | 18 (15.4) | 99 (84.6) |  | 3 (2.6) | 114 (97.4) |  |
| **Occupation** | Housewife | 36 (15.9) | 191 (84.1) | 0.153 | 6 (2.6) | 221 (97.4) | 0.204 |
|  | Formal Job | 10 (27.0) | 27 (73.0) |  | 3 (8.1) | 34 (91.9) |  |
|  | Informal worker/ self employed | 1 (33.3) | 2 (66.7) |  | 0 (0.0) | 3 (100.0) |  |
| **Husband Occupation** | Informal worker/ self employed | 35 (17.7) | 163 (82.3) | 0.999 | 6 (3.0) | 192 (97.0) | 0.466 |
|  | Formal job | 10 (17.5) | 47 (82.5) |  | 2 (3.5) | 55 (96.5) |  |
|  | Non-working | 2 (18.2) | 9 (81.8) |  | 1 (9.1) | 10 (90.9) |  |
| **Displacement** | Yes | 13 (21.0) | 49 (79.0) | 0.546 | 3 (4.8) | 59 (95.2) | 0.349 |
|  | No | 34 (16.6) | 171 (83.4) |  | 6 (2.9) | 199 (97.1) |  |
| **Displacement duration** | Up to a year | 7 (21.1) | 26 (78.8) | 1.000 | 3 (9.1) | 30 (90.9) | 0.144 |
|  | More than a year | 6 (20.7) | 23 (78.3) |  | 0 (0.0) | 29 (100.0) |  |
| **Housing** | Owned house | 28 (16.8) | 139 (83.2) | 0.938 | 6 (3.6) | 161 (96.4) | 0.826 |
|  | Rented house | 3 (20.0) | 12 (80.0) |  | 0 (0.0) | 15 (100.0) |  |
|  | Family or Relatives house | 15 (17.9) | 69 (82.1) |  | 2 (2.4) | 82 (97.6) |  |
| **Household size** | Up to 6 | 22 (13.3) | 143 (86.7) | 0.030 | 5 (3.0) | 160 (97.0) | 0.472 |
|  | More than 6 | 25 (24.5) | 77 (75.5) |  | 4 (3.9) | 98 (96.1) |  |
| **Health Insurance** | None | 27 (17.0) | 132 (83.0) | 0.903 | 4 (2.5) | 155 (97.5) | 0.557 |
|  | SHI | 18 (18.9) | 77 (81.1) |  | 5 (5.3) | 90 (94.7) |  |
|  | Private | 2 (14.4) | 11 (84.6) |  | 1 (0.0) | 13 (100.0) |  |

* Association is significant at the level 0.05 (2-tailed)

Supplementary Table 3. Impact of ANC Use on Birth, Neonatal, and Maternal outcomes at Merowe Locality, Northern state, Sudan 2024. n=267

| **Variable** | **Sub-category** | **Less than 4 visits (%)** | **4 or more visits (%)** | **P-value** | **Less than 8 visits (%)** | **8 or more visits (%)** | **P-value** |
| --- | --- | --- | --- | --- | --- | --- | --- |
| **Maternal Adverse Outcome Incidence** | Yes | 15 (32.6) | 54 (24.5) | 0.342 | 47 (23.9) | 22 (31.9) | 0.250 |
|  | No | 31 (67.4) | 166 (75.5) |  | 150 (76.1) | 47 (68.1) |  |
| **Neonatal Adverse Outcome Incidence** | Yes | 7 (15.2) | 39 (17.7) | 0.845 | 35 (17.8) | 11 (15.9) | 0.873 |
|  | No | 39 (84.8) | 181 (82.3) |  | 162 (82.2) | 58 (84.1) |  |
| **Birth Outcome** | Normal Viable | 42 (91.3) | 216 (98.2) | 0.032* | 191 (97.0) | 67 (97.1) | 1.000 |
|  | Peri-natal death | 4 (8.7) | 4 (1.8) |  | 6 (3.0) | 2 (2.9) |  |
| **Obstructed Labor/ failure to progress** | Yes | 3 (6.5) | 8 (3.6) | 0.292 | 6 (3.0) | 5 (7.2) | 0.160 |
|  | No | 43 (93.5) | 212 (96.4) |  | 64 (92.8) | 191 (97.0) |  |
| **Pre-mature rupture of membrane** | Yes | 0 (0.0) | 7 (3.2) | 0.608 | 4 (2.0) | 3 (4.3) | 0.380 |
|  | No | 46 (100.0) | 213 (96.8) |  | 66 (95.7) | 193 (98.0) |  |
| **Eclampsia and preeclampsia** | Yes | 2 (4.3) | 6 (2.7) | 0.416 | 4 (2.0) | 4 (5.8) | 0.211 |
|  | No | 44 (95.7) | 214 (97.3) |  | 193 (98.0) | 65 (94.2) |  |
| **Gestational diabetes** | Yes | 1 (2.2) | 6 (2.7) | 0.652 | 4 (2.0) | 3 (4.3) | 0.380 |
|  | No | 45 (97.8) | 214 (97.3) |  | 193 (98.0) | 66 (42.9) |  |
| **Gestational HTN** | Yes | 2 (4.3) | 18 (8.2) | 0.292 | 15 (7.6) | 5 (7.2) | 1.000 |
|  | No | 44 (95.7) | 202 (91.8) |  | 182 (92.4) | 64 (92.8) |  |
| **Antepartum or Postpartum Haemorrhage** | Yes | 1 (2.2) | 3 (1.4) | 0.534 | 2 (1.0) | 2 (2.9) | 0.277 |
|  | No | 45 (97.8) | 217 (98.6) |  | 195 (99.0) | 67 (97.1) |  |
| **Maternal ICU admission** | Yes | 0 (0.0) | 2 (0.9) | 1.000 | 2 (1.0) | 0 (0.0) | 1.000 |
|  | No | 46 (100.0) | 218 (99.1) |  | 195 (99.0) | 69 (100.0) |  |
| **Anaemia** | Yes | 6 (13.0) | 20 (9.0) | 0.281 | 20 (10.2) | 6 (8.7) | 0.908 |
|  | No | 40 (87.0) | 200 (90.9) |  | 177 (89.8) | 63 (91.3) |  |
| **UTI in pregnancy** | Yes | 20 (43.5) | 95 (43.2) | 1.000 | 78 (39.6) | 37 (53.6) | 0.060 |
|  | No | 26 (56.5) | 125 (56.8) |  | 119 (60.4) | 32 (46.4) |  |
| **Preterm delivery** | Yes | 4 (8.7) | 22 (10.0) | 1.000 | 21 (10.7) | 5 (7.2) | 0.558 |
|  | No | 42 (91.3) | 198 (90.0) |  | 176 (89.3) | 64 (92.8) |  |
| **LBW** | Yes | 0 (0.0) | 10 (8.9) | 0.356 | 6 (6.5) | 4 (10.8) | 0.469 |
|  | No | 18 (100.0) | 102 (91.1) |  | 87 (93.5) | 33 (89.2) |  |
| **Neonate Small for gestational age** | Yes | 0 (0.0) | 7 (6.2) | 0.592 | 4 (4.3) | 3 (8.1) | 0.402 |
|  | No | 18 (100.0) | 106 (93.8) |  | 90 (95.7) | 34 (91.9) |  |
| **Neonate Large for gestational age** | Yes | 1 (5.6) | 9 (8.0) | 1.000 | 4 (4.3) | 6 (16.2) | 0.031* |
|  | No | 17 (94.4) | 103 (92.0) |  | 89 (95.7) | 31 (83.8) |  |
| **Neonatal sepsis** | Yes | 0 (0.0) | 5 (2.3) | 0.591 | 4 (2.0) | 1 (1.4) | 1.000 |
|  | No | 46 (100.0) | 215 (97.7) |  | 193 (98.0) | 68 (98.6) |  |
| **Neonatal Jaundice** | Yes | 0 (0.0) | 4 (100.0) | 1.000 | 3 (1.5) | 1 (1.4) | 1.000 |
|  | No | 46 (100.0) | 216 (98.2) |  | 194 (98.5) | 68 (98.6) |  |
| **Neonatal respiratory distress** | Yes | 1 (2.2) | 5 (2.3) | 1.000 | 4 (2.0) | 2 (2.9) | 0.651 |
|  | No | 45 (97.8) | 215 (97.7) |  | 193 (98.0) | 67 (97.1) |  |
| **NICU admission** | Yes | 2 (4.3) | 17 (7.7) | 0.543 | 11 (5.6) | 8 (11.6) | 0.107 |
|  | No | 44 (95.7) | 203 (92.3) |  | 186 (94.4) | 61 (88.4) |  |

* Association is significant at the level 0.05 (2-tailed)
